# Peritoneal Cytokines as Early Biomarkers of Colorectal Anastomotic Leakage Following Surgery for Colorectal Cancer: A Meta-Analysis

**DOI:** 10.3389/fonc.2021.791462

**Published:** 2022-01-21

**Authors:** Xin-Yu Qi, Mao-Xing Liu, Kai Xu, Pin Gao, Fei Tan, Zhen-Dan Yao, Nan Zhang, Hong Yang, Cheng-Hai Zhang, Jia-Di Xing, Ming Cui, Xiang-Qian Su

**Affiliations:** Key Laboratory of Carcinogenesis and Translational Research (Ministry of Education), Department of Gastrointestinal Surgery IV, Peking University Cancer Hospital and Institute, Beijing, China

**Keywords:** peritoneal fluid, cytokines, anastomotic leakage, biomarkers, colorectal cancer

## Abstract

**Background:**

Postoperative colorectal anastomotic leakage (CAL) is a devastating complication following colorectal resection. However, the diagnosis of anastomotic leakage is often delayed because the current methods of identification are unable to achieve 100% clinical sensitivity and specificity. This meta-analysis aimed to evaluate the predictive value of peritoneal fluid cytokines in the detection of CAL following colorectal surgery.

**Methods:**

A comprehensive search was conducted on PubMed, Embase, Cochrane Library, and Web of Science before June 2021 to retrieve studies regarding peritoneal fluid cytokines as early markers of CAL. Pooled analyses of interleukin (IL)-1β, IL-6, IL-10, and tumor necrosis factor (TNF) were performed. The means (MD) and standard deviations (SD) of the peritoneal fluid cytokines were extracted from the included studies. Review Manager Software 5.3 was used for data analysis.

**Results:**

We included eight studies with 580 patients, among which 85 (14.7%) and 522 (44.5%) were evaluated as the CAL and non-CAL groups, respectively. Compared to the non-CAL group, the CAL group had significantly higher peritoneal IL-6 levels on postoperative day (POD) 1–3 (P = 0.0006, 0.0002, and 0.002, respectively) and slightly higher TNF levels on POD 4 (P = 0.0002). Peritoneal levels of IL-1β and IL-10 were not significantly different between the two groups in this study.

**Conclusion:**

Peritoneal IL-6 levels can be a diagnostic marker for CAL following colorectal surgery, whereas the value of TNF needs further exploration in the future.

**Systematic Review Registration:**

[https://www.crd.york.ac.uk/prospero/#myprospero], PROSPERO (CRD42021274973)

## Introduction

Postoperative colorectal anastomotic leakage (CAL) is a devastating complication occurring in 1%–20% of cases after colorectal surgery (1). It is associated with increased costs, in-patient time, and in-hospital mortality, and reoperation may also be needed (2). A recent meta-analysis (3) further demonstrated that anastomotic leakage was associated with poor oncologic prognosis, including increased local recurrence and decreased overall survival, cancer-specific survival, and disease-free survival. Currently, the detection of anastomotic leakage mainly relies on computer tomography (CT) and some nonspecific laboratory indicators such as increased leukocyte, C-reactive protein (CRP), and procalcitonin levels. Around 20% of anastomotic leakage cases are usually diagnosed at a mean of 6–15 days after discharge (4). Unfortunately, a retrospective study found that a 2.5-day delay in the detection of anastomotic leakage increased mortality rates from 24% to 39% (5), which means that many patients with early-stage CAL are left undetected until significant disease progression. Thus, the current diagnostic strategies have an obvious lag and have a difficulty in identifying CAL in the early stage. Therefore, the early detection of CAL is of clinical great importance.

As an inflammatory biomarker, cytokines in the drain fluid have been suggested as an effective method for the early identification of CAL. With respect to the value of peritoneal fluid cytokines, two meta-analyses by Cini et al. (6) and Sparreboom et al. (7) demonstrated that increased interleukin (IL)-6 and tumor necrosis factor (TNF)-α levels in the drain fluid were related to CAL and might contribute to its early detection. However, these meta-analyses were published before 2016 and were not registered on the International Prospective Register of Systematic Review (PROSPERO), and these simply used the random-effects model in their data analysis without further discussing the cause of higher heterogeneity in their study.

The controversial conclusions and lack of previous meta-analyses necessitate further exploration with a larger sample size and more rigorous statistical analysis. This meta-analysis aims to further explore the predictive value of peritoneal fluid cytokines in the detection of CAL following colorectal surgery. We present the following article/case in accordance with the PRISMA reporting checklist.

## Materials and Methods

This study has been registered and published on PROSPERO (CRD42021274973), and it was conducted in line with the Preferred Reporting Items for Systematic Reviews and Meta-Analyses (PRISMA) guidelines (8).

### Data Sources

A comprehensive search of PubMed, Cochrane Library, Embase, and Web of Science was performed by three authors independently. Combinations of subject words and free words related to the cytokines of CAL were used for literature search. The following keywords were used: colorectal neoplasm, colorectal tumors, colorectal cancer, colorectal carcinoma, anastomotic leaks, anastomotic leakage, and cytokine. The related articles function was also used in this study.

### Inclusion and Exclusion Criteria

The inclusion criteria were as follows: (1) a full paper regarding peritoneal cytokines of CAL in English, (2) comparison between CAL and non-CAL patients in the same study, (3) the latest or higher quality literature in cases of duplicate published data, and (4) evaluation of at least one out of four outcomes of interest (i.e., peritoneal levels of IL-1β, IL-6, IL-10, and TNF). Nonhuman studies, congress abstracts, case reports, and letters were excluded.

### Methodological Quality of Included Studies

Three authors independently used ROBINS-I to evaluate the included studies according to seven aspects, including bias due to confounding, bias in the selection of participants into the study, bias in the classification of interventions, bias due to deviations from intended interventions, bias due to missing data, bias in the measurement of outcomes, and bias in the selection of the reported results. Only the study of Oikonomakis et al. (9) was evaluated as high risk, and the other studies were assessed as moderate risk, as shown in **Table 1**.

**Table 1:** Quality assessment of non-randomized using risk of bias in nonrandomized studies of interventions.

ROBINS-I	Bias due to confounding	Bias in selection of participants into the study	Bias in classification of interventions	Bias due to deviations from intended interventions	Bias due to missing data	Bias in measurement of outcomes	Bias in selection of the reported result	Overall
Herwig et al.	M	M	L	L	L	L	M	M
Bertram et al.	M	L	L	L	L	L	L	M
Matthiessen et al.	M	L	L	L	L	L	L	M
Yamamoto et al.	L	L	L	L	L	L	L	M
Fouda et al.	M	L	L	L	L	L	L	M
Bilgin et al.	M	L	L	L	L	L	L	M
Sparreboom et al.	L	L	L	L	M	L	L	M
Oikonomakis et al.	M	S	M	L	L	L	S	S

ROBINS-I, risk of bias in nonrandomized studies of interventions; S, serious; M, moderate; L, lower.

### Data Extraction

Data were extracted independently by three authors. Any discrepancies were resolved by repeat evaluation until reaching an agreement. We recorded the following information: first author, year of publication, country, study type, inclusion period, sample size, and outcomes of interest. The outcomes of interest was CAL and the included parameters were peritoneal cytokines: IL-1β, IL-6, IL-10, and TNF. The means (MD) and standard deviations (SD) of the cytokine levels per POD were extracted. Although the articles of Yamamoto et al. (10) and Matthiessen et al. (11) did not report the MD and SD of the cytokine levels per POD, the meta-analysis of Sparreboom et al. (7) contained the detailed data of these two studies. All data on cytokines were switched to the same unit (ng/mL) in this study. For incomplete or missing data, the primary authors of each study were contacted for related data, but nothing was provided.

### Definitions

The definitions of CAL were inconsistent among the included studies, as shown in **Table 2**. Matthiessen et al. (11), Fouda et al. (15), and Bilgin et al. (14) defined CAL according to the clinical signs, especially in terms of drain fluid. However, Herwig et al. (12) and Sparreboom et al. (16) determined CAL mostly based on imaging evaluation, such as abdominal CT scan. The study of Bertram et al. (13) was dependent on laparotomy, whereas the study of Yamamoto et al. (10) focused on both clinical signs and imaging evaluation. All included studies used ELISA or CLIA to measure cytokine levels, as shown in **Table 3**.

**Table 2:** Definition of anastomotic leakage of included studies.

Author	Year	Definition
Herwig et al. (12)	2002	Diagnosis of AL was confirmed by endoscopy, contrast enema, abdominal CT scan, microbiologic examination, and finall intraoperative findings during relaparotomy.
Bertram et al. (13)	2003	Patients were considered uneventful if recovery occurred without signs of anastomotic leakage within 14 days after operation. Anastomotic leakage was confirmed by laparotomy.
Matthiessen et al. (11)	2007	The definition of anastomotic leakage in this study was clinical: peritonitis caused by leakage, pelvic abscess, discharge of feces from the abdominal drain, or rectovaginal fistula, and leakage from all staple lines were included.
Yamamoto et al. (10)	2011	The diagnosis of postoperative peritonitis was made on the basis of the clinical findings along with the imaging data and the colour of abdominal exudates in the drainage tube.
Fouda et al. (15)	2011	AL was defined clinically as gas, pus, or fecal discharge from the drain, fecal discharge from the operative wound, pelvic abscess, peritonitis, and rectovaginal fistul.
Bilgin et al. (14)	2017	A suspicion for an anastomotic leakage occurred when the patient had fever after the post-operative third day, existence of fecal or suspicious fluid coming from the drain was observed, or the patient had abdominal tenderness.
Sparreboom et al. (16)	2020	AL was confirmed by either endoscopy, CT scan and/or contrast enema or reoperation. Fistulas communicating with the anastomosis on CT scan were classified as AL together with presacral abscesses if extravasation of the colonic contrast was visible on radiological imaging.
Oikonomakis et al. (9)	2019	Not specifically described.

AL, anastomotic leakage; CT, computed tomography.

**Table 3:** Methodology of cytokine level measurement of included studies.

Author	Year	Cytokines	Measuring	Company
Herwig et al. (12)	2002	IL-1β, IL-6, TNF	ELISA	Coulter-Immunotech Diagnostics, Hamburg, Germany
Bertram et al. (13)	2003	IL-6, TNF	CLIA	Immulite, DPC Biermann GmbH, Bad Nauheim, Germany
Matthiessen et al. (11)	2007	TNF, Il-6, IL-10	CLIA	DPC, Los Angeles, CA
Yamamoto et al. (10)	2011	IL-1β, IL-6, TNF	ELISA	R&D system, Minneapolis, MN, USA
Fouda et al. (15)	2011	TNF, Il-6, IL-10	ELISA	Not mentioned
Bilgin et al. (14)	2017	IL-6, TNF	ELISA	Eastbiopharm Co. Ltd, Hangzhou
Sparreboom et al. (16)	2020	IL-1β,IL-6,IL-10,TNF	ELISA	Thermo Fisher Scientific, Bleiswijk, The Netherlands
Oikonomakis et al. (9)	2019	IL-6, IL-10	CLIA	DPC, Los Angeles, California,IL, USA

IL-1β, interleukin 1-beta; IL-6, interleukin-6; IL-10, interleukin-10; TNF, tumour necrosis factor; ELISA, enzyme linked immunosorbent assay; CLIA, chemiluminescence analysis.

### Statistical Analysis

Statistical analyses were performed using Review Manager Software 5.3 (The Cochrane Collaboration, London, UK). Quantitative data were described as their MD with their 95% confidence intervals (95%CI). Quantitative data were presented as the median with range or quartile, and the mean and standard deviation were calculated based on previously described methods (17). Statistical heterogeneity was evaluated using chi-square test and *I^2^* statistics, which reflects the percentage of variation in study estimates due to heterogeneity. A random-effects model was used when *I^2^* > 50%, which indicated higher heterogeneity. Otherwise, the fixed-effect model was used for analysis. In cases wherein the outcomes of interest had high heterogeneity, a sensitivity analysis was performed to analyze the causes of heterogeneity. The quality of nonrandomized controlled trials was evaluated using the Risk of Bias in Non-randomized Studies of Interventions (ROBINS-I) (18).

## Results

### Description of Eligible Studies

**Figure 1:**
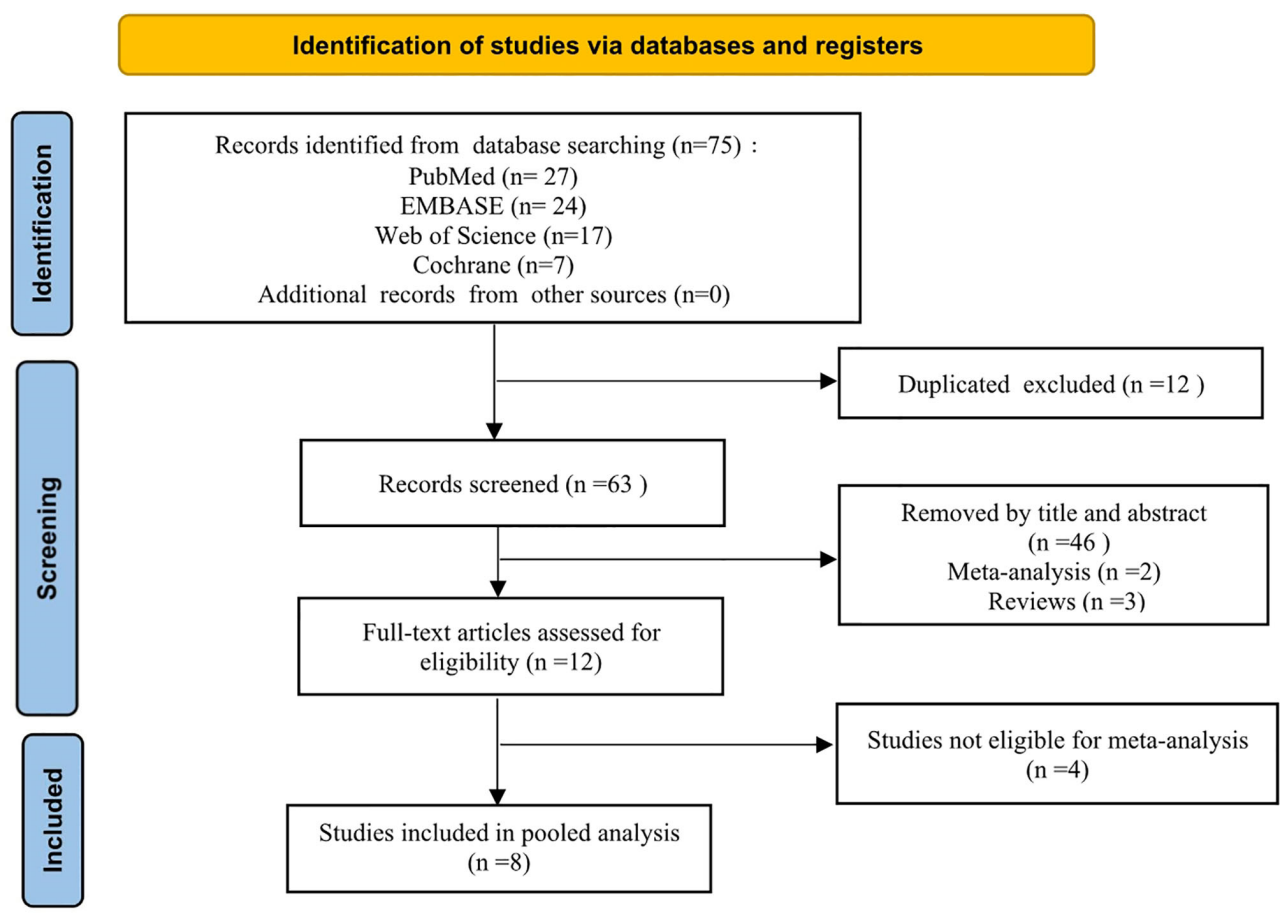
Flow diagram of the meta-analysis literature search and study selection.

A PRISMA flowchart showing the selection of studies for this meta-analysis is presented in **Figure 1**. In total, eight studies (9–16) published from 2002 to 2019 met the inclusion criteria, and their characteristics are shown in **Table 4**. The most recent study retrieved under the search strategy was published on October 19, 2019. These eight studies had a total of 580 patients (85 CAL and 495 non-CAL) and included one multicenter (16) and seven single-center (9–15) studies.

**Table 4:** Characteristics of included studies.

Author	Year	Country	Study type	Inclusion period	Group	No. of Patients	Surgery type	Outcome of interest	Follow-up (days)
Herwig et al. (12)	2002	Australia	P	1996.12 -1997.12	CAL	12	Colorectal surgery	IL-1β, IL-6, TNF	9
					Non-CAL	12			
Bertram et al. (13)	2003	Germany	P	2001.03 - 2001.09	CAL	3	Colorectal surgery	IL-6, TNF	7
					Non-CAL	22			
Matthiessen et al. (11)	2007	Sweden	P	2002.11 - 2004.10	CAL	4	Anterior resection	TNF, Il-6, IL-10	6
					Non-CAL	19			
Yamamoto et al. (10)	2011	Japan	P	Unclear	CAL	8	Colorectal surgery	IL-1β, IL-6, TNF	3
					Non-CAL	92			
Fouda et al. (15)	2011	Egypt	P	2007.03 - 2009.12	CAL	8	Low anterior resection	TNF, Il-6, IL-10	5
					Non-CAL	48			
Bilgin et al. (14)	2017	Turkey	Case-control	2012.03 - 2013.04	CAL	7	Low anterior resection	IL-6, TNF	5
					Non-CAL	43			
Sparreboom et al. (16)	2020	Netherland	P	2015.08 - 2017.10	CAL	38	Rectal surgery	IL-1β,IL-6,IL-10,TNF	3
					Non-CAL	254			
Oikonomakis et al. (9)	2019	Sweden	Case-control	Unclear	CAL	7	Low anterior resection	IL-6, IL-10	7
					Non-CAL	13			

IL-1β, interleukin 1-beta; IL-6, interleukin-6; IL-10, interleukin-10; TNF, tumour necrosis factor; P, prospective; CAL, colorectal anastomotic leakage; Non-CAL, None colorectal anastomotic Leakage.

Although the studies of Ugraset al. (19) and Alonso et al. (20) met the inclusion criteria, they were not included in the analysis because the peritoneal cytokine levels reported by Ugraset al. (19) were 10–1000 times higher than those in the included studies, and those reported by Alonso et al. (20) were 50–100 times lower. Normally, a meta-analysis should compare similar data to obtain more accurate results, but the data of these two studies are so heterogeneous compared to the other studies that they cannot be analyzed together. This was also reported in the meta-analysis of Sparreboom et al. (7).

### Meta-Analysis of Peritoneal Cytokines

**Figure 2:**
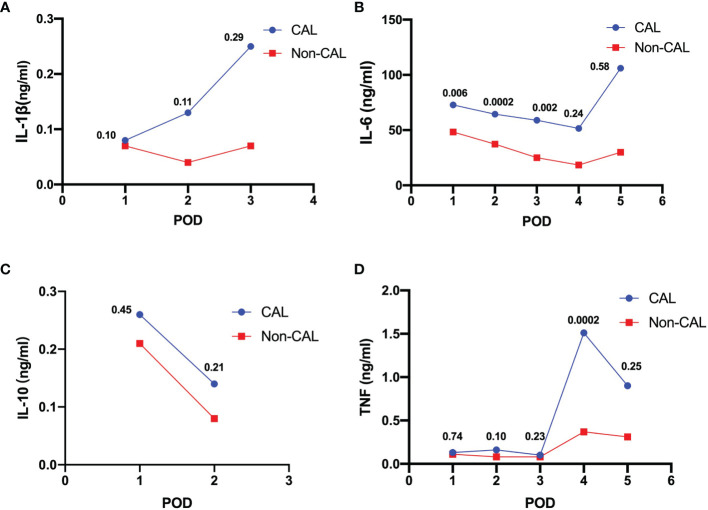
Weighted means of peritoneal levels of IL-1β (**A**, ng/mL), IL-6 (**B**, ng/mL), IL-10 (**C**, ng/mL) and TNF (**D**, ng/mL) on patients with CAL and non-CAL patients each postoperative day. The *P* values of statistical significance are marked when relevant.

The mean levels of peritoneal cytokines on each POD were recorded by calculating the weighted mean of the included studies, as shown in **Figure 2**. IL-1β, IL-6, and TNF, but not IL-10, gradually increased in the CAL group versus the non-CAL group.

#### Interleukin-1β

**Figure 3:**
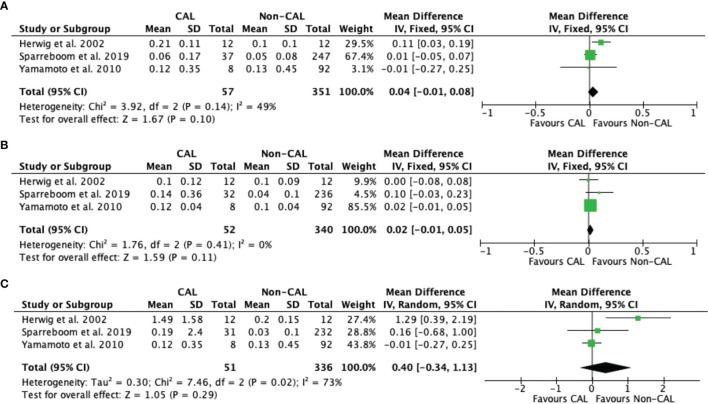
Forest plot of peritoneal levels of IL-1β (ng/mL) on patients with CAL and non-CAL patients each postoperative days (POD) 1 **(A)**, 2 **(B)**, and 3 **(C)**.

Pooled data from three studies (10, 12, 16) revealed that IL-1β levels were not significantly different between CAL and non-CAL patients on POD 1 (MD: 0.04, 95%CI: −0.01–0.08, *P* = 0.1) (**Figure 3A**), POD 2 (MD: 0.02, 95%CI: −0.01–0.05, *P* = 0.11) (**Figure 3B**), and POD 3 (MD: 0.40, 95%CI: −0.34–1.13, *P* = 0.29) (**Figure 3C**), with a fixed-effect model during analysis.

#### Interleukin-6

**Figure 4:**
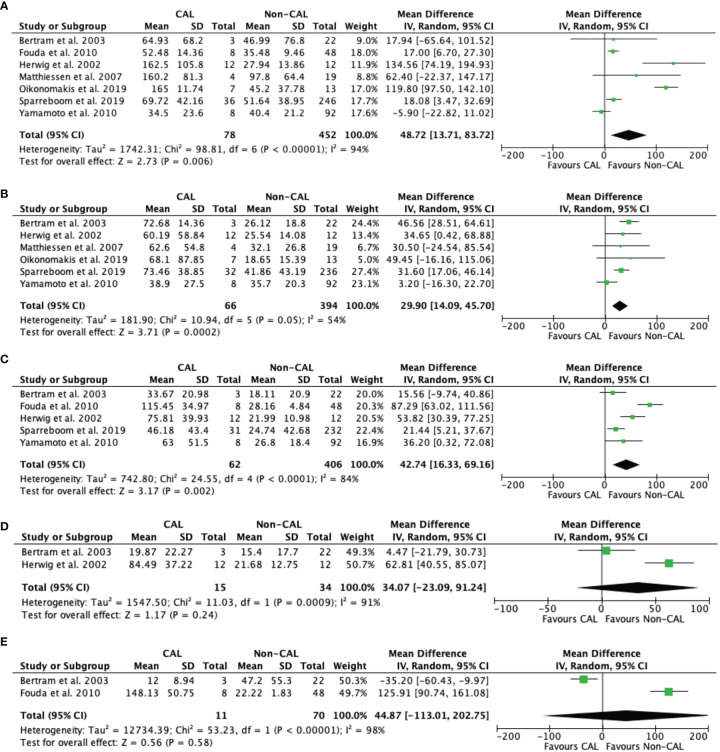
Forest plot of peritoneal levels of IL-6 (ng/mL) on patients with CAL and non-CAL patients each postoperative days (POD) 1 **(A)**, 2 **(B)**, 3 **(C)**, 4 **(D)** and 5 **(E)**.

Pooled data from seven studies (9–13, 15, 16) revealed that CAL patients had significantly higher IL-6 levels versus non-CAL patients on POD 1 (MD: 48.72, 95%CI: 13.71–83.72, *P* = 0.006) with high heterogeneity (*P* < 0.001, *I^2^* = 94%) (**Figure 4A**). Pooled data from six studies (9–13, 16), and five studies (10, 12, 13, 15, 16) also revealed that CAL patients had significantly higher IL-6 levels versus non-CAL patients on POD 2 (MD: 29.90, 95%CI: 14.09–45.70, *P* = 0.0002) with high heterogeneity (*P* = 0.05, *I^2^* = 54%) (**Figure 4B**) and POD 3 (MD: 42.74, 95%CI: 16.33–69.16, *P* = 0.002) with high heterogeneity (*P* < 0.001, *I^2^* = 84%) (**Figure 4C**), respectively. No significant difference was found between CAL and non-CAL patients on POD 4 (MD: 34.07, 95%CI: −23.09–91.24, *P* = 0.24) (**Figure 4D**) and POD 5 (MD: 44.87, 95%CI: −113.01–202.75, *P* = 0.58) (**Figure 4E**).

#### Interleukin-10

**Figure 5:**
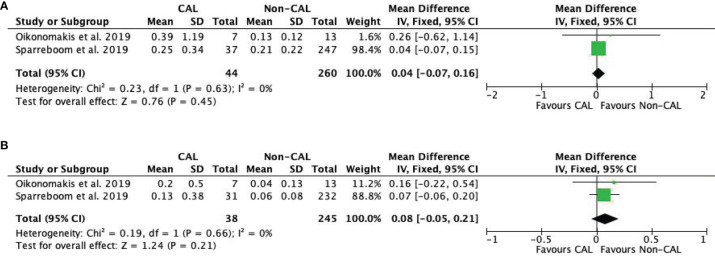
Forest plot of peritoneal levels of IL-10 (ng/mL) on patients with CAL and non-CAL patients each postoperative days (POD) 1 **(A)** and 2 **(B)**.

Pooled data from two studies (9, 16) revealed that IL-10 levels were not significantly different between CAL and non-CAL patients on POD 1 (MD: 0.04, 95%CI: −0.07–0.16, *P* = 0.45) (**Figure 5A**) and POD 2 (MD: 0.08, 95%CI: −0.05–0.21, *P* = 0.21) (**Figure 5B**) with a fixed-effect model.

#### Tumor Necrosis Factor

**Figure 6:**
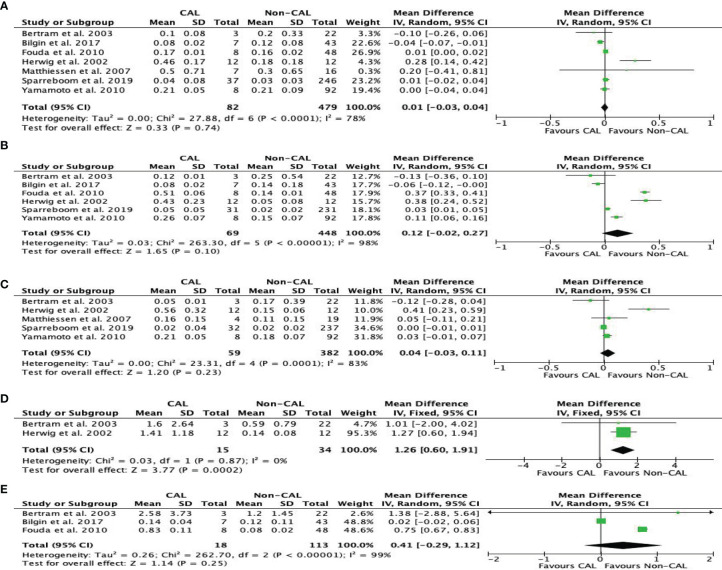
Forest plot of peritoneal levels of TNF (ng/mL) on patients with CAL and non-CAL patients each postoperative days (POD) 1 **(A)**, 2 **(B)**, 3 **(C)**, 4 **(D)** and 5 **(E)**.

TNF levels were reported by seven studies (10–16). Our meta-analysis found that CAL patients had higher peritoneal TNF levels than non-CAL patients on POD 4 (MD: 1.26, 95%CI: 0.60–1.91, *P* = 0.0002) (**Figure 6D**) with a fixed-effect model. TNF was not significantly different between CAL and non-CAL patients on POD 1 (MD: 0.01, 95%CI: −0.03–0.04, *P* = 0.74) (**Figure 6A**), POD 2 (MD: 0.12, 95%CI: −0.02–0.27, *P* = 0.10) (**Figure 6B**), POD 3 (MD: 0.04, 95 CI: −0.03–0.11, *P* = 0.23) (**Figure 6C**), and POD 5 (MD: 0.41, 95%CI: −0.29–1.12, *P* = 0.25) (**Figure 6E**) with a random-effects model.

### Sensitivity Analysis

Because the results of studies involving IL-6 and TNF were highly heterogeneous, a sensitive analysis was conducted to analyze the causes of heterogeneity. The studies of Herwig et al. (12) and Oikonomakis et al. (9) were the main drivers of heterogeneity in terms of peritoneal TNF and IL-6, respectively. Removing these studies (9, 12) from their respective groups caused the heterogeneity to significantly decline, but the results of the meta-analysis remained unchanged, further confirming the reliability of the conclusion.

## Discussion

CAL after colorectal surgery is a serious complication that can lead to severe infection, and thus, it is critical to identify this in its early stages. The potential clinical factors related to CAL have been widely reported. A high-quality meta-analysis (21) involving in 14 studies demonstrated that male gender, BMI≥25kg/m2, ASA score>2, tumor size >5 cm and preoperative chemotherapy were associated with the development of AL. A recent meta-analysis (22) in surgical related risk factor of AL successively reported that patients with no defunctioning stoma and intraoperative blood transfusion had a higher incidence of AL following surgery. Meanwhile, biomarkers for early diagnosis of CAL have attracted more and more attention. Cytokines such as IL-1, IL-6, IL-10, and TNF-α are polypeptides with known roles in the immune response (23). Wiik et al. (24) reported that the concentrations of all measured cytokines were enormously higher locally at the operative site than in the systemic circulation. Similarly, Jansson et al. (25) also demonstrated that compared with systemic cytokines, the measurement of peritoneal cytokines is more sensible for determining postoperative inflammatory reactions. Yamamoto et al. (10) found that peritoneal levels of TNF-α, IL-6, and IL-1β on postoperative day (POD) 3 may be an additional early diagnostic predictor of intra-abdominal complications following colorectal surgery. The recent study of Sparreboom et al. (16) involving the largest sample size so far identified only peritoneal TNF-α on POD 1 as part of the prediction model based on multivariate penalized logistic regression. Thus, our meta-analysis further investigated the previous studies focused on the early identification of CAL by measuring the peritoneal fluid cytokines. We found that the peritoneal levels of cytokines, such as IL-6, were higher among CAL patients versus non-CAL patients on POD 1–3, implying the potential of IL-6 level as an early diagnostic marker of CAL following colorectal surgery.

An experimental animal study found that systemic IL-6 administration has a direct detrimental effect on the healing of colonic anastomoses (26). In this meta-analysis, high peritoneal levels of IL-6 correlated with CAL on POD 1–3, which was consistent with the results of the previous two meta-analyses (6, 7). Several previous studies (27–29) found that elevated IL-1β, IL-6, and TNF-α levels were associated with surgical stress, including length of operation, hemorrhage, and high peritoneal bacterial counts. In response to surgical trauma, these cytokines are mainly secreted by macrophages and are released from the surgical site within the first few hours after surgery. Patients who recover uneventfully have low or even decreased levels of cytokines in the drainage fluid within 24 h after surgery. Moreover, several studies found that patients with increased peritoneal levels of IL-6 and TNF-α on POD 1 are prone to anastomotic leakage (11, 12, 19). On the other hand, the third largest study of Yamamoto et al. (10) with 100 patients who underwent left-side resection demonstrated no significant difference in IL-6 levels until POD 3, whereas Bertram et al. (13) even reported no significant difference in peritoneal IL-6 and TNF-α within 7 days after surgery. However, it is worth nothing that the lower incidence of anastomotic leakage (8%) in the study of Yamamoto et al. (10) and the small sample size (25 patients) in the study of Bertram et al. (13) made it inappropriate to statistically investigate the difference in their studies. Moreover, the inconsistent inclusion criteria and definitions of anastomotic leakage could potentially affect the results. Meanwhile, a recent case–control study (20) with 60 patients further explored the level of IL-6 in serum and drainage on POD 2 and 4 following surgery. That study reported increased peritoneal IL-6 levels on POD 2 and 4, and serum levels were only significantly different starting on the 4th day. This result further proved the value of peritoneal IL-6 level in the recognition of anastomotic leakage. Moreover, the peritoneal IL-6 level was measured at values 10 times higher compared to TNF-α. This might be due to the long half-life of IL-6, making it a better marker of CAL following surgery. Combined with clinical symptoms, signs and radiological findings, peritoneal levels of IL-6, as a supplement to inflammatory factors, it contributed to improve the early diagnosis of CAL. Moreover, Dulk et al. (5) reported that a 2.5-day delay in lacking anastomotic leakage-specific treatments might increase mortality from 24% to 39%. Although the 8 original studies included didn’t mention appropriate and timely treatments in detail, we believe that timely application of antibiotics and puncture drainage will help improve the patient’s prognosis when the CAL was diagnosed following surgery.

In this study, peritoneal TNF level barely had statistical significance on POD 4 after colorectal surgery. By contrast, the previous meta-analysis of Sparreboom (7) reported that CAL patients had significantly higher peritoneal TNF levels versus non-CAL patients on POD 3–5. Mowever, Yamamoto et al. (10) and Fouda et al. (15) successively reported that the peritoneal level of TNF-α was not statistically significant until POD 3. Both studies (13, 15) found that the TNF-α levels of non-CAL patients decreased from POD 1 to POD 3 following surgery. Upon careful review of these two studies, the timing of cytokine measurement was different between the two, which may cause some results to be missed. The study of Fouda et al. (15) mainly focused on POD 1, 3, and 5, whereas that of Yamamoto (10) focused on POD 1–3. In addition, the level of TNF-α, as the first cytokine to increase after sepsis or trauma, might vary greatly because of its short half-life in the peritoneal fluid, making it unreliable at certain times. Although its presence has been detected in the pelvic fluid after colorectal surgery, it only shows that it is a local reaction at the microscopic level after surgery. Bertram et al. (13) reported that TNF-α level didn’t increase significantly until the day of operative demonstration of anastomotic leakage. Despite the rise of TNF-α was found, this still did not reach a statistical difference. When the postoperative recovery is uneventful, the levels of TNF level gradually decrease in theory. However, patients without AL also showed an upward trend of TNF-α when comparing with those out, this situation may not be fully explained by experimental technical problems (13). Furthermore, with more original studies are included, the stability of the conclusions has changed in this study. Therefore, it is still debatable whether TNF-α can be used as a marker, and more studies with a larger sample size are needed to further explore its significance.

Consistent with a previous meta-analysis (7), IL-1β and IL-10 had no significant difference between CAL and non-CAL patients in this study. An animal study by Poll (30) demonstrated that endogenous IL-10 protects mice from death during septic peritonitis. Therefore, IL-10 as an anti-inflammatory factor plays an impotent role in weakening the inflammatory process (31). In our meta-analysis, peritoneal levels of IL-1β and IL-10 were only reported by 2–3 studies. More definitive results could have been achieved if more studies were included. The meta-analysis of Cini (6) concluded that the measurement of drain fluid cytokines had diagnostic potential for preclinical-stage CAL, but it also recognized that further research is needed, possibly using a combination of cytokines as markers. The second largest published study by Sammour (32) with 206 patients who underwent colorectal surgery demonstrated that the peritoneal levels of IL-6 and IL-10 on POD 1 were predictive of anastomotic leakage (area under receiver operating characteristic curve: 0.72 and 0.74; *P* = 0.006 and 0.004, respectively). Similar studies are needed for further discussion in the future. On the other hand, the recent largest multicenter study published by Sparreboom (16), with 292 patients undergoing rectal cancer resection, found that the combination of serum CRP and peritoneal matrix metalloproteinase-9 (MMP-9), rather than peritoneal cytokines, might be useful for the early prediction of anastomotic leakage on POD 3. Therefore, other biomarkers such as growth factors, neopterin, and kynurenine are also worthy of further exploration.

In contrast to the results of a recent meta-analysis (7), our study was only able to identify increased peritoneal levels of IL-6 as a potential diagnostic marker of CAL following colorectal surgery.The study does have several limitations. First, owing to the lower rate of anastomotic leakage following colorectal surgery, studies with a larger sample size and higher-level evidence are difficult to develop. Using rigorous statistical methods such as sensitivity analysis, potential problems, especially selection bias, might affect the reliability of this study. Second, the small sample size, inconsistent timing of sample measurement, and various diagnostic criteria for anastomotic leakage across the included studies would further weaken the reliability of the results in this study. Third, the discriminative value of peritoneal cytokines remains unclear, and this was also an important deficiency of the included studies. Therefore, studies with higher-level evidence are needed to further explore the role of peritoneal cytokines in the early diagnosis of CAL.

## Conclusion

The peritoneal level of IL-6 has potential as a diagnostic marker of CAL following colorectal surgery, whereas the value of TNF-α needs further exploration in the future.

## Data Availability Statement

The original contributions presented in the study are included in the article/supplementary material. Further inquiries can be directed to the corresponding authors.

## Author Contributions

KX, M-XL, and X-YQ participated in the acquisition, analysis, and interpretation of data, as well as in the manuscript drafting. PG and FT participated in data acquisition. Z-DY and NZ participated in analysis and interpretation of data. X-QS and MC contributed to the conception, design, and data interpretation.J-DX, HY, and C-HZ revised the manuscript for important intellectual content. The authors read and approved the final manuscript.

## Funding

This study was supported by the National Natural Science Foundation of China (No. 82171720, 81872022, 81672439), Beijing Natural Science Foundation (No.7162039), Capital’s Funds for Health Improvement and Research (CFH 2018-2-2153), Beijing Hospitals Authority Clinical Medicine Development of Special Funding Support (No. ZYLX202116), Beijing Municipal Administration of Hospitals Incubating Program (No.PX 2016018) and Beijing Excellent Talent Training Funding (2018000021469G258).

## Conflict of Interest

The authors declare that the research was conducted in the absence of any commercial or financial relationships that could be construed as a potential conflict of interest.

## Publisher’s Note

All claims expressed in this article are solely those of the authors and do not necessarily represent those of their affiliated organizations, or those of the publisher, the editors and the reviewers. Any product that may be evaluated in this article, or claim that may be made by its manufacturer, is not guaranteed or endorsed by the publisher.
